# Styptic Wool

**Published:** 1871-03

**Authors:** 


### STYPTIC WOOL.

Dr. Ehrle, of Isny, prepares this by boiling the finest carded cotton wool for half an hour or an hour in a solution containing four per cent. of soda, then thoroughly washes it out in cold spring water, wrings and dries it. The wool is thus effectually purified, and is now capable of imbibing fluids uniformly. It is then to be dipped two or three times in fluid chloride of iron diluted with one-third of water, expressed and dried in a draught of air, but not in the sun or with the aid of high heat; finally it is carded out. Thus prepared, it is of a beautiful yellowish-brown color, and feels like ordinary dry cotton wool. As it is highly hygroscopic, it must be kept dry, and when required to be transported must be packed in caoutchouc or bladder. Charpie may be prepared in a similar manner, but, on account of its coarser texture, is not so effective as cotton wool, presenting a less surface for producing coagulation. When the wool is placed on a bleeding wound, it induces moderate contraction of the tissue, coagulation of the blood that has escaped, and subsequently coagulation of the blood that is contained within the injured vessels, and thus arrests the hemorrhage. The coagulating power of the chloride of iron is clearly exalted by the extension of its surface that is in this way effected. The application of the prepared wool is not particularly painful, whilst, by sucking up the superfluous discharge, and preventing its decomposition, it seems to operate favorably on the progress of the wound. The unpleasant secondary results that have led many practiced surgeons to discard the use of the perchloride of iron do not occur with the wool when it is properly made and applied. In cases of wounds where the bleeding proceeds from large and deep-seated vessels, it may be used as a compress, a bandage being applied over it.

or the wound may be plugged with it. It may also be employed with advantage in cases of profuse suppuration, to imbibe the discharge and purify the surface. He recommends that a small portion should be given to every soldier on going into action.—*Lancet.*

